# Examination of Differences in Nonmedical Supplemental Benefit Coverage for Dual-Eligible Enrollees in Medicare Advantage Plans in 2021

**DOI:** 10.1001/jamanetworkopen.2022.35161

**Published:** 2022-10-06

**Authors:** Bonnie Jin, Lingshu Xue, John Lovelace, Donna Almario Doebler, Eric T. Roberts

**Affiliations:** 1 Department of Health Policy and Management, University of Pittsburgh School of Public Health, Pittsburgh, Pennsylvania; 2 UPMC Center for High-Value Health Care, University of Pittsburgh Medical Center, Pittsburgh, Pennsylvania

## Abstract

This cross-sectional study uses Medicare Advantage benefit package data for 2021 to examine differences in the coverage of nonmedical supplemental benefits—such as transportation services and food and meal assistance—for dual-eligible enrollees with health-related social needs.

## Introduction

The 2017 Creating High-Quality Results and Outcomes Necessary to Improve Chronic Care Act, alongside other regulatory changes, has given Medicare Advantage (MA) plans flexibility to offer nonmedical supplemental benefits (eg, transportation services, food and meal assistance) to address health-related social needs.^1,2^ Some MA plans, known as dual-eligible special needs plans (D-SNPs), exclusively serve Medicare beneficiaries who are dually eligible for Medicaid; such plans play an important role because dual-eligible beneficiaries tend to experience a high burden of chronic and disabling conditions and health-related social needs.^3^ A subset of plans, known as fully integrated dual-eligible SNPs (FIDE-SNPs), cover Medicare and Medicaid spending and thus have stronger incentives to cover supplemental benefits that reduce spending.^4^

Little is known about the extent to which D-SNPs and FIDE-SNPs offer nonmedical supplemental benefits, which benefits are covered, or how the mix of covered benefits differs from general MA plans that do not exclusively serve dual-eligible beneficiaries. We examined how coverage of these benefits differed for dual-eligible beneficiaries enrolled in D-SNPs, FIDE-SNPs, and general MA plans in 2021.

## Methods

This cross-sectional study used public MA benefit package data linked to national counts of full-benefit dually eligible enrollees by contract and plan benefit package for 2021. Because we analyzed deidentified data, the University of Pittsburgh Institutional Review Board deemed this study exempt and informed consent was waived. The study followed the STROBE reporting guideline.

Here, the term *plan* refers to each unique combination of MA contract and plan benefit package. We estimated the number and percentage of dual-eligible beneficiaries enrolled in plans offering nonmedical supplemental benefits overall and by type of supplemental benefit. Statistical analysis was conducted with SAS version 9 (SAS Institute Inc).

## Results

According to our analysis, 3 665 019 dual-eligible beneficiaries were enrolled in MA plans in 2021. Of these, 60.5% were enrolled in D-SNPs, 7.8% were enrolled in FIDE-SNPs, and 31.7% were enrolled in general MA plans (Table). Of the 59 FIDE-SNPs, 25 (42.4%) offered at least 1 nonmedical supplemental benefit, accounting for 63.8% of dual-eligible enrollees. Furthermore, 150 of 485 D-SNPs (30.9%) and 451 of 3089 general MA plans (14.6%) offered at least 1 nonmedical supplemental benefit, accounting for 22.9% and 19.9% of dual-eligible enrollees, respectively.

**Table.** Enrollment in and Number of Medicare Advantage Plans Offering Nonmedical Supplemental Benefits by Plan Type in 2021

Parameter	Dual-eligible special needs plans (n = 485)	Fully integrated dual-eligible special needs plans (n = 59)	General Medicare Advantage plans (n = 3089)
No. of enrollees (%)^a^	2 217 691 (60.5)	285 696 (7.8)	1 161 632 (31.7)
No. of enrollees offered ≥1 nonmedical supplemental benefit	507 734 (22.9)	182 377 (63.8)	230 657 (19.9)
No. of plans offering ≥1 nonmedical supplemental benefit^b^	150 (30.9)	25 (42.4)	451 (14.6)
Ten most commonly offered benefits, No. of enrollees (%)			
Food and produce^c^	320 023 (14.4)	118 359 (41.4)	102 133 (8.8)
Pest control	225 346 (10.2)	742 (0.3)	67 297 (5.8)
Transportation for nonmedical needs	188 789 (8.5)	44 365 (15.5)	56 579 (4.9)
Home-delivered meals	177 235 (8.0)	8535 (3.0)	86 052 (7.4)
Indoor air-quality equipment and services	156 145 (7.0)	34 (<0.1)	20 393 (1.8)
Social needs benefit (for individuals with chronic illnesses)^d^	117 319 (5.3)	5027 (1.8)	43 506 (3.7)
Service dog support	139 671 (6.3)	131 (<0.1)	17 760 (1.5)
Home and bathroom safety devices and modifications	108 150 (4.9)	14 597 (5.1)	18 641 (1.6)
General support for living^e^	95 709 (4.3)	34 (<0.1)	21 107 (1.8)
Services supporting self-direction^f^	85 514 (3.9)	5027 (1.8)	17 237 (1.5)

^a^ Numbers of full-benefit dually eligible beneficiaries enrolled in plans based on Medicare administrative enrollment data for January 2021 are presented. Shares of partial-benefit dual-eligible beneficiaries enrolled in Medicare Advantage plans were not included.

^b^ Plans were defined as unique combinations of Medicare Advantage contract and plan benefit package identifiers.

^c^ Food and produce includes benefits provided to assist enrollees in meeting nutritional needs, such as fresh produce, frozen foods, and canned goods.

^d^ Examples include access to community or plan-sponsored programs or events to address social needs, such as club memberships, park passes, family counseling, and companion care.

^e^ Examples include plan-sponsored housing consultations and subsidies for rent and utilities.

^f^ Services include those meant to allow enrollees to have responsibility for managing all aspects of their health care delivery such as establishing power of attorney, taking financial literacy classes, and accessing interpreter services.

Among FIDE-SNP beneficiaries, 41.4% were enrolled in plans covering food and produce benefits and 15.5% were offered nonmedical transportation services (Table). Among dual-eligible enrollees, 14.4% in D-SNPs and 8.8% in general MA plans were offered food and produce benefits. Nonmedical transportation was covered for 8.5% of dual-eligible enrollees in D-SNPs and for 5.8% in general MA plans. Benefits such as home-delivered meals, home modifications, and social services for individuals with chronic illnesses were offered to less than 10.0% of dual-eligible beneficiaries. Of the 42 supplemental benefits available, 22 were offered to less than 1.0% of dual-eligible beneficiaries; 8 were not offered by any plan (Box).

Box. **Ten Least Commonly Offered Nonmedical Supplemental Benefits Across All Medicare Advantage Plans and Benefits Not Covered by Any Plan**

**Least commonly offered nonmedical benefits across all Medicare Advantage plan types**

Pet care services
Data plan
In-home safety assessment
Thorough house cleaning
Independence and safe mobility with AAA
Nutritional/dietary benefit
Health education
Virtual visit
Social needs benefit
Grocery delivery coverage

**Benefits not covered by any Medicare Advantage plan**

Additional smoking and tobacco cessation counseling sessions
Complementary therapies
Counseling services
Enhanced disease management
Postdischarge in-home medication reconciliation
Readmission prevention
Weight management programs
Wigs for chemotherapy-related hair loss

## Discussion

This cross-sectional study found that MA plans exclusively serving dual-eligible beneficiaries offered more nonmedical supplemental benefits than general MA plans in 2021. However, this coverage was low, particularly among D-SNPs. Although FIDE-SNPs were more likely to cover these benefits, they accounted for only 7.8% of total enrollment across all plan types. The most common benefits were for food and produce, meal delivery, and nonmedical transportation. Home care services and other social services were available to fewer dual-eligible beneficiaries across all plan types. These findings suggest that MA plans serving these beneficiaries have an opportunity to expand coverage of supplemental nonhealth benefits.

One study limitation is that we only assessed the availability of supplemental benefits and not their utilization rates, which may be lower. Furthermore, we could not examine the overlap of benefits offered by MA plans and Medicaid.^5^ Increased monitoring of the availability and use of these benefits is needed to determine whether MA plans that serve dual-eligible beneficiaries are enhancing services for vulnerable populations.^6^ Greater financial alignment across MA and Medicaid plans may increase benefit coverage by plans.
